# Supplementary material for: Polymorphisms in Pfkelch13 domains before and after the introduction of artemisinin-based combination therapy in Southwest Nigeria
Source: PLoS One. 2025 Mar 31;20(3):e0316479. doi: 10.1371/journal.pone.0316479 (PMC11957316; doi:10.1371/journal.pone.0316479)

Samples: 12320
Bases: 1036
Average spacing: 12

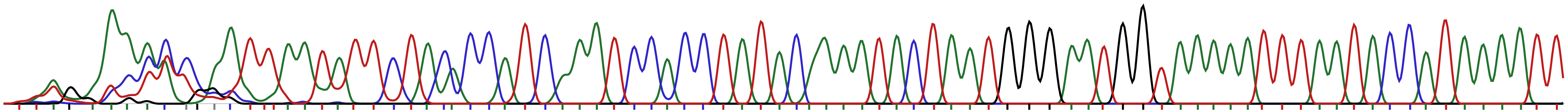

T T A G A A A A C - G - C T T T A A T A T T C T A C A C C A T C A A A T C C A C C T A T A C A A A A T A C T A A T G G G A A T G G T A A A A A T T T A A T A C C A T A A A A T T

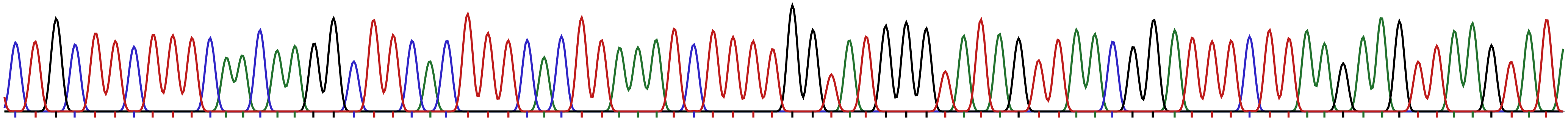

C T G C T T C T T T C A A C A A G G C T T C A C T T T C A C T T A A A T C T T T T G G T A T G G G T A T A G T T A A C G G A T T T C T T A A G A A G T T A A G T A T

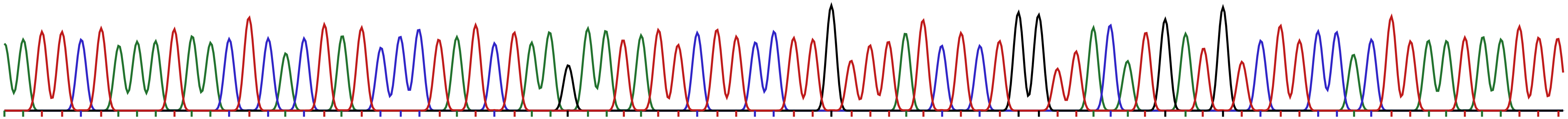

A A T T C T A A A T A A C T C A C T A T C C C T A T C T A A G A A T A T T C T T C C T T G T T T A T C T C T G G T T A C A T G A T G T C T T C C A C T T A A T A A T T T

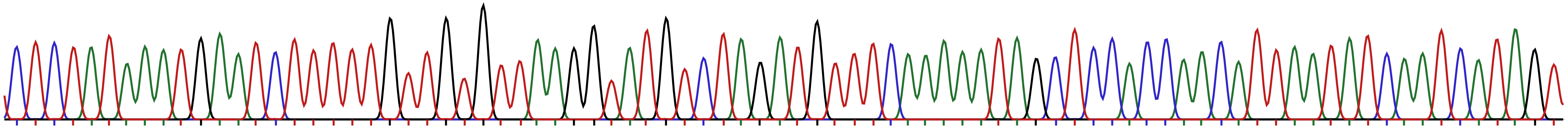

C T C T A T A A A T G A A T C T T T T T G T T G T G T T A A G G T A T G T C T A G A T G T T T C A A A A A T A G C T C C A C C A A C A T T A A T A T C A A T C A T A G T

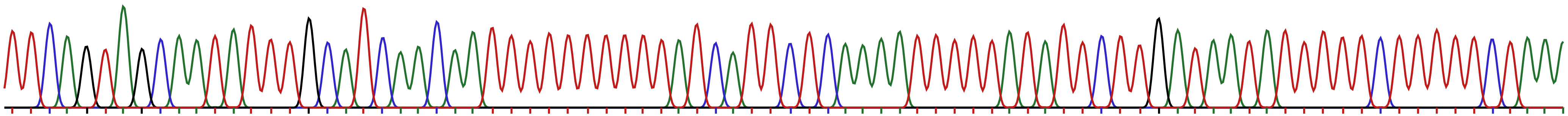

T T C A G T A G C A A T A T T T G C A T C A A C A A T T T T T T T T T T A T C A T T C T C A A A A T T T T T A T A T T C T T G A T A A T A T T T T T C T T T T T C T A A A

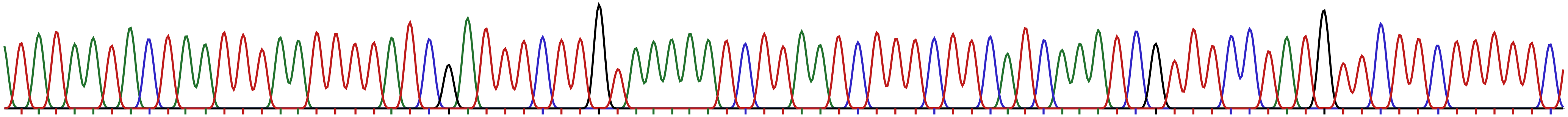

T A T A A T A C T A A T T T A A T T T T A T C G A T T T C T T G T A A A A A T C T T A A T C T T T C T T C A T C A A A T C G T T T C C T A T G T T C T T C T T T T T C

Samples: 12320
Bases: 1036
Average spacing: 12



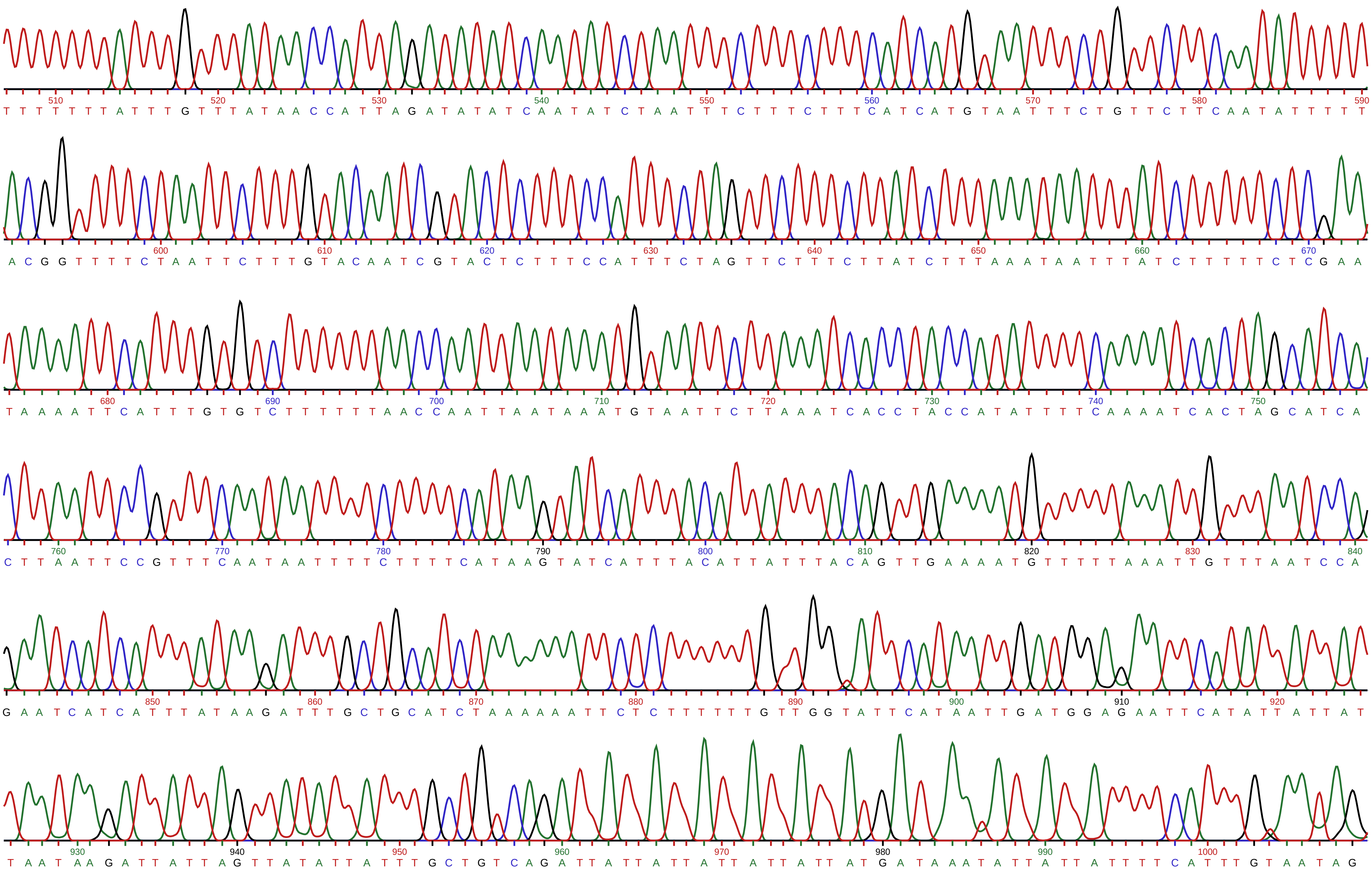

Samples: 12320
Bases: 1036
Average spacing: 12



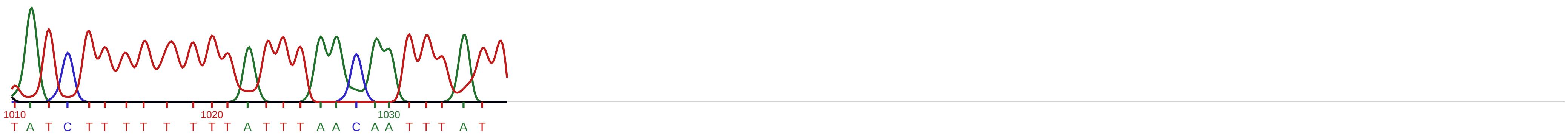

Supplement: Supporting information 2 — (ZIP) [file pone.0316479.s002.zip › 076KN2R_PREMIX_Plate_KELCH2_G11.pdf]
